# Themes Underlying Australian General Practitioner Views towards Chiropractic and Osteopathy: An Assessment of Free Text Data from a Cross-Sectional Survey

**DOI:** 10.1155/2018/2786106

**Published:** 2018-01-14

**Authors:** Sandra Grace, Roger Engel, Ian Jalsion

**Affiliations:** ^1^School of Health & Human Sciences, Southern Cross University, Military Road, Lismore, NSW 2480, Australia; ^2^Department of Chiropractic, Macquarie University, Balaclava Road, North Ryde, NSW 2109, Australia

## Abstract

The Australian chiropractic and osteopathic professions underwent a period of significant transformation between 1960 and 2000. This resulted in an improvement in the views held by the medical profession towards the two professions. However, a recent survey of Australian general practitioners (GPs) reported that a number of GPs still hold negative views towards chiropractors and osteopaths. This paper examines these views from the perspective of critical realism and explores the generative mechanisms that can influence the willingness of health practitioners to collaborate over patient care. A qualitative analysis of open-ended responses to a survey of 630 Australian GPs was conducted. Unfavourable attitudes of GPs towards chiropractors and osteopaths included perceived lack of safety, efficacy, and inadequacy of training, despite chiropractic's and osteopathy's reliance on the same evidence base and similar training to those of other manual therapy professions such as physiotherapy. These attitudes may be underpinned by the professional biases against chiropractic and osteopathy that continue to marginalise the professions within the Australian healthcare system. Continued investment in the research base for chiropractic and osteopathic practice is required, along with raising the awareness of GPs about the education and skills of chiropractors and osteopaths.

## 1. Introduction

While both chiropractic and osteopathy are formally recognised as allied health professions, their role within the Australian healthcare system has not been clearly delineated [1]. This may in part be the result of attitudes of Australian general medical practitioners (GPs) towards the two professions.

Between 1960 and 2000, the chiropractic and osteopathic professions in Australia underwent a period of significant transformation which included practitioner registration, the creation of codes of practice, and an upgrade in educational standards [2–7]. The period was also characterised by the amalgamation of a number of smaller professional associations to form larger associations [8]. Inclusion of chiropractic and osteopathic services on the Australian Government's Medicare Benefits Schedule was granted in 2005 [9] and the professions were designated as allied health professions soon after [10].

These changes brought a shift in attitude by sections of the medical profession in favour of chiropractic and osteopathy. Some GPs were willing to refer patients for chiropractic and osteopathic treatment in an attempt to collaborate over patient care [11–13]. Despite the shift in attitude, the chiropractic and osteopathic professions failed to grasp the opportunity to expand their research base through collaborative projects. This may have been due to a lack of research training and funding within the professions [14] but also to the view held by some about the methodological difficulties associated with identifying appropriate placebo controls for testing complex, individualised interventions such as chiropractic and osteopathy [15, 16]. This failure to increase the level of research left the professions open to criticism and scepticism from mainstream medicine.

A recent survey of 630 Australian GPs about their views towards chiropractors and osteopaths reported that a number of GPs still hold negative views towards chiropractors and osteopaths [1]. While the results from this study raised awareness of GP attitudes within the chiropractic profession [17], they did not improve understanding about the nature of the dissatisfaction. Strongly held negative views about chiropractic and osteopathy have the potential to influence discussions with patients about treatment choices. Given the increasing demand for chiropractic and osteopathic services, improving the understanding of GPs about these professions may help to meet the demands of a healthcare system that is becoming increasingly patient-centred and reliant on healthcare teams. The aim of this paper is to examine attitudes of Australian GPs towards chiropractic and osteopathy from the perspective of critical realism. This approach explores the generative mechanisms that may underpin such attitudes.

## 2. Methods

**Figure 1:**
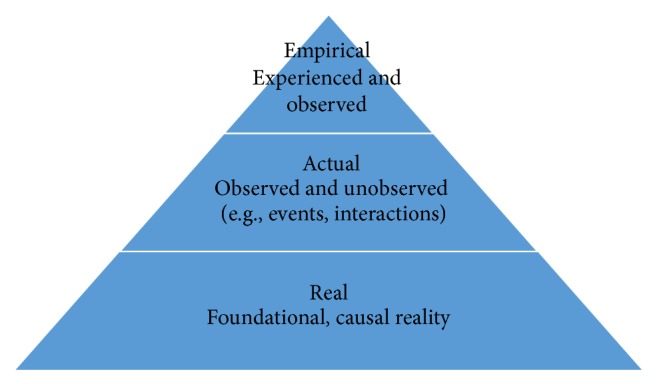
The stratified ontological framework of critical realism.

Critical realism, first described by Bhaskar [18], is a philosophy that can accommodate the complexity of our world by examining it from multiple perspectives. It combines “mind-independence” (the existence of the real world independent of human experience) with social construction (the interpretation of the real world according to our own experiences and life histories). It provides an “ontological and epistemological position from which to research people in their social/health context” [19]. Critical realism is characterised by a stratified ontological framework: the “empirical” and most superficial level is observable and experiential but it may be an incomplete understanding of the phenomenon under examination; the “actual” level includes both the observed and unobserved aspects of our world (e.g., the people, objects, interactions, and events in our world) [20]; and the “real” or deepest level that underpins these. It is from the “real” ontological level that we can identify generative mechanisms or tendencies that influence the actual and empirical worlds we inhabit (see Figure 1).

An assessment of free text responses from a cross-sectional study designed to investigate the current views of Australian GPs towards chiropractic and osteopathy was conducted from the perspective of this stratified ontology. The cross-sectional study targeted GPs currently working in Australian private practice and used an anonymous online survey to gather data. Prior to the survey, the questions were tested on a focus group of 19 experienced vocational trainers attending a regional GP training conference. The survey link was promoted at a national GP conference followed by publication on a national association website and electronic publications and advertorials on a national commercial website for Australian doctors. The response rate for the survey was 2.6%. While this rate limits the generalisability of the data, the themes generated by the qualitative analysis should still be considered trustworthy. Comparison between the demographics of respondents in the cross-sectional study and the relevant workforce data showed a reasonably good correlation for gender and location by state. However, a greater proportion of respondents were over 40 years of age with less than 10 years in practice compared to the national average making the current cohort somewhat different to the national profile [1].

The survey contained a total of 43 questions, 39 of which were structured as closed-ended questions with options provided. Two questions were open-ended and provided space for free text and two questions were mixed (i.e., options were provided plus the opportunity to add free text). The results reported here are from the four questions that included provision for free text. Free text responses provided a rich data set. They were independently read and re-read by the researchers (SG, RE, and IJ) to identify initial patterns or conceptual codes. Through an iterative process of comparing, refining, and coalescing codes, higher order themes were identified and continually refined until consensus was reached [21].

Statistical analyses were performed on the themes from the responses to the four questions involving free text responses; pairs of questions relating to chiropractic and osteopathy were analysed using McNemar's exact test for dependent binary data [22]. A level of *α* = 0.05 was set as the threshold for statistical significance. The statistical software R [R Core Team 2017] was used together with the “exact2x2” package [22] for all analyses.

All respondents consented to participate in the online survey. The study was approved by Macquarie University's Human Research Ethics Committee (approval number: 5201400200).

## 3. Results

One hundred and eighty-four (184) GPs responded to both the chiropractic and osteopathic questions. Five themes emerged from an analysis of these responses. The themes were often strongly expressed, as demonstrated by quotations from the free texts. Unfavourable responses towards chiropractic outnumbered those towards osteopathy with significant differences between the two professions reported below.

### 3.1. Theme 1: Chiropractic Is Not a Safe Practice

The first theme to emerge in the free text responses was that chiropractic was not a safe practice. A total of 24 out of 184 (13.0%) respondents raised concerns about the potentially harmful side effects associated with chiropractic but not osteopathic intervention. Only 2 out of 184 (1.1%) respondents raised similar concerns about osteopathic but not chiropractic treatment while 2/184 (1.1%) raised concerns about both. McNemar's Test: estimated odds of concern about harm in osteopathic versus chiropractic were 0.08 (95% CI: 0.01, 0.34; *p* < 0.001). Typical comments included the following:

I have had patients with serious detrimental outcomes following these “treatments.”

I have seen patients firsthand that have had bad results at the hands of a chiropractor.

Some of my patients have had vertebral artery dissections from chiropractic treatment. I am aware of patients who have died from this complication.

### 3.2. Theme 2: There Is a Lack of Evidence to Support Chiropractic and Osteopathy

The second theme to emerge in the free text responses was a lack of evidence to support either chiropractic or osteopathy. Just over one fifth of respondents (40 out of 184; 21.7%) referred to the lack of evidence for the efficacy of chiropractic intervention while almost the same proportion (35 out of 184; 19.0%) raised a similar concern with respect to the efficacy of osteopathic intervention. Of these respondents, 21/184 were concerned with the efficacy of both practitioner types (11.4%). McNemar's Test: estimated odds of concern about efficacy in osteopathic versus chiropractic were 0.74 (95% CI: 0.34, 0.55; *p* = 0.74).

Although 59 out of 492 responses (12%) reported that chiropractic and osteopathy offered real benefits, especially when dealing with musculoskeletal issues, these benefits were thought to be largely dependent on a patient's belief in these professions (i.e., the placebo effect). Typical comments included the following:

I do not believe there is any evidence for chiropractic treatment and there are unnecessary risks involved.

Limited evidence base for chiropractor treatment effectiveness. Need proper medical peer-reviewed studies.

I only refer to evidence-based allied health practitioners.

### 3.3. Theme 3: Chiropractors and Osteopaths Are Insufficiently Trained and Should Not Be Allowed to Practise as Primary Contact Health Practitioners

The third theme to emerge in the free text responses was the inadequacy of chiropractic and osteopathy education programs. Overall, respondents were apprehensive about the fitness of graduates with a Master's degree in either discipline to practise as primary contact health practitioners. A total of 48 out of 184 respondents (24.5%) raised the issue of credibility with regard to the education background of chiropractors, while 28 out of 184 (15.2%) raised the same concern about osteopathic education. Among these respondents, 23/184 (12.5%) raised concerns about both chiropractic and osteopathic education. McNemar's Test: estimated odds of concern about osteopathic versus chiropractic education were 0.23 (95% CI: 0.07, 0.62; *p* = 0.002).

Included as part of this theme was the issue of variability in treatment approaches within the professions. A total of 19 out of 184 responses (10.3%) referred to an unwillingness to refer patients to chiropractors due to an inability to identify “good” from “bad” chiropractors. Only 7 out of 184 respondents (3.8%) raised similar concerns about osteopaths. Among these respondents, 2 were concerned about referring to either practitioner type. McNemar's Test: estimated odds of concern about referring to osteopath versus chiropractor were 0.29 (95% CI: 0.08, 0.83; *p* = 0.017). Typical comments included the following:

Lack of trust in their ability to effectively manage a patient's pain and its aetiology.

I keep getting stupid letters from chiropractors about my patients that indicate chiros are practising well beyond their scope of practice. For example, one chiro informed me he had done a thorough assessment and diagnosed the patient's problem as their “adrenal meridians” were out of alignment.

Why would I? It uses a system of imaginary structures like the “innate” to treat real disease. It's make-believe.

The issue of chiropractors' and osteopaths' views on vaccination was also raised: 24 out of 184 respondents (13.0%) identified the antivaccination stance that some chiropractors uphold as problematic; 2 of these respondents (1.1%) had the same concern about osteopaths while none of the respondents were concerned about osteopaths but not chiropractors. McNemar's Test: estimated odds of concern about antivaccination policies for osteopaths versus chiropractors were 0 (95% CI: 0, 0.18; *p* < 0.001). Typical comments included the following:

There are also a number of chiropractors who openly advocate against vaccination, and I can't support this.

Whilst there seems to be some evidence for spinal mobilisation in the short term treatment of back pain, it's difficult to know which practitioners will stick to the evidence and which will be hosting seminars on the evils of immunisation.

I don't believe in the science behind chiro and I am very concerned about reported inappropriate treatments and advice people get from chiropractors, especially regarding childhood health and immunisation.

### 3.4. Theme 4: Chiropractors Make Financially Motivated Decisions about Treatment That Are Associated with False Advertising

The fourth theme to emerge in the free text responses was the issue of unprofessional practices. Just under one-third (59 out of 184; 32.1%) of the responses referred to concerns about chiropractors making financially motivated decisions and their use of false claims about the benefits of chiropractic intervention in their advertising. Only 13 out of 184 responses (7.1%) raised similar concerns about osteopathy. Among these, 10 respondents were concerned about both osteopaths and chiropractors motivations. McNemar's Test: estimated odds of concern about osteopaths versus chiropractors were 0.06 (95% CI: 0.01, 0.19; *p* < 0.001).

Typical comments included the following:

Too many patients describe inappropriate, if not negligent personal experiences, especially patients who are vulnerable due to financial or other social distress. For example, one parent reported their chiropractor recommended years of weekly therapy for their child with developmental delay.

They [chiropractors] have dubious and expensive treatment plans. Also they almost always mislead patients and give false information. They inappropriately order X-rays and then withhold X-rays so we never see them.

It's exploitative in that ongoing treatment is encouraged, and chiropractors have ventured far out of the musculoskeletal realm, often promoting dangerous pseudoscience such as vaccine refusal. They also “treat” healthy babies and claim completely unscientific powers - such as spinal manipulation as a treatment for colic and ear infections.

### 3.5. Theme 5: Chiropractic and Osteopathy Are Redundant due to the Presence of Physiotherapy

The fifth theme to emerge from the free text responses was that chiropractors and osteopaths were duplicating physiotherapy services. As physiotherapy was regarded as evidence-based and safe by many respondents, GPs could refer patients to physiotherapists for treatment of musculoskeletal conditions, thereby making chiropractic and osteopathic services redundant. Typical comments included the following:

Too risky. No reason to need this kind of treatment. We have physios.

I think physio has more evidence base for efficacy.

I feel that physios use better evidence base and [have] a more sound theoretical basis of their modalities.

I believe there are alternative allied health professionals that can provide more appropriate treatments (e.g. physiotherapy).

## 4. Discussion

The findings of this study reveal that GPs' negative attitudes towards chiropractic and osteopathy were based on a perceived lack of safety and efficacy, perceived inadequacy of educational programs, questionable practices of some practitioners, and the failure of both professions to promote their contributions to healthcare. From the perspective of critical realism, these perceptions are located at the empirical level; that is, they are based on GPs' observations and experiences. The views of GPs towards chiropractors were generally less favourable than towards osteopaths. These negative views raise concerns for both professions as they have the potential to impact government policy and budget determinations.

In critical realism, the “empirical” level does not tell the whole story; the “actual” ontological level comprises the unobservable as well as the observable and experiential [19]. It includes actual objects, events, people, interactions with GPs, chiropractors, osteopaths, and patients, interactions between these groups, and both the observable and unobservable evidence underpinning practice.

While safety should always be a primary concern when considering an intervention, a recent systematic review on the use of nonpharmacological therapies for low back pain reported continuing support for the use of spinal manipulation in managing this common condition [23]. Furthermore, new evidence showing that some pharmacological interventions commonly used to treat low back pain and sciatica are no more effective than nonpharmacological interventions, yet carry known risk of addiction and side effects, highlights the question of equity in assessing safety [24, 25]. While such findings do not excuse chiropractic and osteopathic researchers from having to produce evidence of safety and efficacy, it appears that some GPs may not be aware of current evidence for the management of musculoskeletal conditions (this is their reality based on their experience). For them, a crucial part of this evidence is “unobservable” or hidden from view (the “actual” ontological level).

All chiropractic and osteopathic training programs in Australia are university-based [6]. The comments about educational standards reported in this survey do not question* where* the education occurs but rather its adequacy in preparing primary contact health practitioners. This is based on their experience and observation (the “empirical” ontological level). What appears to be hidden from the view of some GPs (the “actual” ontological level) is that all accredited chiropractic, osteopathy, and physiotherapy curricula in Australia are designed to produce primary contact health practitioners who have patients that can present with previously undiagnosed health conditions. The training ensures that graduates have the necessary knowledge and skills for appropriate evidence-based management of musculoskeletal conditions and referral procedures where applicable. Consistent with the proposed interpretation of the data, improving GPs' knowledge about the level and quality of training of chiropractors and osteopaths may be indicated. Of concern is the general lack of interest by GPs to learn more about chiropractic and osteopathic education: 68% of GPs reported a lack of interest in chiropractic education and 63% in osteopathic education [1].

Observed or experienced (“empirical”) questionable practices fall within the realm of a profession's Code of Conduct which, in Australia, is under the jurisdiction of the relevant professional regulatory body, in this case the Chiropractic and Osteopathy Boards of Australia. At the “actual” ontological level, both boards have revised their respective Codes of Conduct. However, the number of complaints about questionable practices and unsubstantiated claims of efficacy continues to rise [26, 27].

The issue of relevance to the current Australian healthcare system continues to receive little attention from both the chiropractic and osteopathic professions in Australia. By not clearly defining their respective roles, we believe that both professions have failed to represent their respective contributions to the health of the Australian community. GPs often see the duplication of musculoskeletal services as confusing and want to simplify the message to their patients by referring to a single group, in this case physiotherapists. This is their experience and observation (“empirical” ontological level). Moreover, health policy makers could claim that a duplication of services leads to higher costs and recommend referring to one group on the basis of budgetary impact alone. Chiropractors and osteopaths argue that their professions are distinct from one another as well as being different from physiotherapy and other manual therapies [28]. However, it is clear that these professions have a lot in common, including a number of diagnostic and treatment techniques (the “actual” ontological level) [29]. To date, research has focused to a large extent on identifying distinctive world views which could influence clinical reasoning and treatment design [30].

### 4.1. Difference in Opinions towards the Professions

While there were significant differences in the views of GPs towards each profession, these differences may simply be a product of the size of the workforce: as of June 2016 there were approximately two and a half times more chiropractors than osteopaths in Australia (chiropractors: 5167; osteopaths: 2094) so there are more chances for GPs to interact with chiropractors than with osteopaths [31]. This argument is supported by evidence that chiropractors see more patients on average than osteopaths, compounding the effect of a disparity in practitioner numbers [32]. The difference in attitudes could also be a product of different approaches to practice, with chiropractors appearing to adopt a more aggressive marketing approach to patient management compared to osteopaths. Support for this argument includes comments reported in this paper under Theme 4: financially motivated decisions about treatment. Furthermore, a recent report claimed that over 35% of chiropractic treatments in one jurisdiction in Australia were provided for “wellness” or “maintenance” care [33]. These treatments have come to be regarded as preventative in nature. However, there is currently little evidence to support such a view. The claim that chiropractors and osteopaths are practising outside their scope of practice is difficult to assess as the relevant authority, the Australian Health Practitioner Regulation Agency, does not publish data on scope of practice violations for either profession [34].

A number of generative mechanisms emerged from the data analysis. These are the deep-seated mechanisms (causative factors and tendencies) that can influence our perceptions of the “actual” and the “empirical” and can include personal and professional values and institutional discrimination. In this analysis, some apparent professional biases were identified:

(i) While it is understandable that GPs may not be aware that the minimum level of chiropractic and osteopathic training is at Master's degree level, nor be fully acquainted with the content of the curricula, the general lack of interest by GPs in learning more about chiropractic and osteopathic education could be considered alarming (68% of GPs reported a lack of interest in learning about chiropractic education and 63% in osteopathic education) [1] and raises the possibility of professional bias. If all health practitioners are obliged to be familiar with the range of healthcare options available to their patients in order to make informed recommendations and decisions about treatment, then GPs are not exempt from this fundamental requirement for patient-centred care.

(ii) Many GPs in the survey stated their preference for physiotherapy over chiropractic and osteopathy even though both professions use similar diagnostic and treatment techniques [30]. A clear understanding of chiropractic and osteopathic practice and how they compare with physiotherapy practice is called for. There is a growing body of research investigating the effectiveness of physiotherapy care (e.g., manual therapy and exercise compared to patient advice for chronic nonspecific low back pain [35]), but there has been little comparison between physiotherapy and other manual therapies. It appears that some GPs require further education in the range of healthcare practitioners who deliver evidence-based treatments for musculoskeletal conditions.

(iii) Attitudes and behaviours of members of the chiropractic and osteopathy professions may have contributed to the observed and experienced reality of GPs. Both the chiropractic and osteopathic professions need to take responsibility for instances of alleged unprofessional conduct. Certain chiropractors and osteopaths avow their unorthodoxy in the belief that their practice transcends the accepted constraints of evidence-based practice. This bias derives from a philosophical standpoint and is supported by anecdotal claims of clinical success. Such behaviour should not be supported as it represents an attempt to circumvent the scientific process.

### 4.2. Jurisdictional Variations

The views of Australian GPs towards chiropractors and osteopaths are somewhat different to other jurisdictions. In the US, osteopathy has been considered part of mainstream medicine since the 1960s, with osteopaths awarded full MD status in all 50 states. Chiropractic continues to gain recognition with an increasing number of chiropractors appointed under the Veterans Affairs health system. In Canada and the UK, chiropractors work alongside medical practitioners in public hospitals and multidisciplinary medical centres [36], while, in Norway, high referral rates from GPs to chiropractors have been credited with improving patient outcomes and reducing costs associated with treating certain musculoskeletal conditions [36, 37].

### 4.3. Limitations

There are a number of limitations associated with this qualitative analysis. Apart from the issue of sample size which has been addressed previously [1], the majority of GPs who participated in the survey had never referred a patient to either profession (chiropractic: 60%; osteopathy: 66%) and were not interested in learning more about the education of either profession (chiropractors: 68%; osteopaths: 63%). These low levels of interaction and interest may have influenced the respondents' comments and resulted in a proportion of the comments based on opinion rather than fact or experience.

## 5. Conclusion

Negative views towards chiropractic and osteopathy are evident among some Australian GPs. These views centre on five themes: perceived lack of safety and efficacy, perceived inadequacy of training as primary contact health practitioners, questionable practice motives of some practitioners, and relevance to the Australian healthcare system. These attitudes may be the result of ignorance and/or bias on the part of GPs, a situation that has the potential to perpetuate the marginalisation of chiropractors and osteopaths within the Australian healthcare system.
